# Contraindications to the Initiation of Veno-Venous ECMO for Severe Acute Respiratory Failure in Adults: A Systematic Review and Practical Approach Based on the Current Literature

**DOI:** 10.3390/membranes11080584

**Published:** 2021-07-30

**Authors:** Lars-Olav Harnisch, Onnen Moerer

**Affiliations:** Department of Anesthesiology, University Medical Center, University of Goettingen, 37075 Göttingen, Germany; omoerer@med.uni-goettingen.de

**Keywords:** ARDS, acute respiratory distress syndrome, ECMO, extracorporeal membrane oxygenation, contraindication, indication

## Abstract

(1) Background: Extracorporeal membrane oxygenation (ECMO) is increasingly used for acute respiratory failure with few absolute but many relative contraindications. The provider in charge often has a difficult time weighing indications and contraindications to anticipate if the patient will benefit from this treatment, a decision that often decides life and death for the patient. To assist in this process in coming to a good evidence-based decision, we reviewed the available literature. (2) Methods: We performed a systematic review through a literature search of the MEDLINE database of former and current absolute and relative contraindications to the initiation of ECMO treatment. (3) Results: The following relative and absolute contraindications were identified in the literature: absolute—refusal of the use of extracorporeal techniques by the patient, advanced stage of cancer, fatal intracerebral hemorrhage/cerebral herniation/intractable intracranial hypertension, irreversible destruction of the lung parenchyma without the possibility of transplantation, and contraindications to lung transplantation; relative—advanced age, immunosuppressed patients/pharmacological immunosuppression, injurious ventilator settings > 7 days, right-heart failure, hematologic malignancies, especially bone marrow transplantation and graft-versus-host disease, SAPS II score ≥ 60 points, SOFA score > 12 points, PRESERVE score ≥ 5 points, RESP score ≤ −2 points, PRESET score ≥ 6 points, and “do not attempt resuscitation” order (DN(A)R status). (4) Conclusions: We provide a simple-to-follow algorithm that incorporates absolute and relative contraindications to the initiation of ECMO treatment. This algorithm attempts to weigh pros and cons regarding the benefit for an individual patient and hopefully assists caregivers to make better, informed decisions.

## 1. Introduction

The use of veno-venous extracorporeal membrane oxygenation (vvECMO) has gained worldwide acceptance and is today recommended in international guidelines [1,2] as a salvage therapy in patients with severe acute respiratory failure, when conservative measures are unsuccessful. Several studies confirmed a positive effect of ECMO treatment compared with conservative treatment [3,4,5].

Most recently, a meta-analysis of individual patient data from two of the aforementioned studies demonstrated a survival advantage in the group of patients treated with ECMO [6]. These studies have obviously led physicians to using this technique earlier [7], and the number of extracorporeal techniques used worldwide is subsequently increasing [8,9].

Furthermore, this increase is supported by improvements in ECMO devices and systems that not only simplify use, but lead to improved biocompatibility through improved circuit materials and their composition [10,11,12,13,14]. With the technical issues largely resolved and increasing experience in clinical management, the biggest challenge today is selecting the right candidates for vvECMO, i.e., those who will benefit most from this invasive treatment.

To identify these patients, it is necessary to consider indications and contraindications for ECMO initiation. The contraindications to the initiation of ECMO therapy are not uniformly agreed upon, and each center, as well as each provider involved in the indication for the initiation of ECMO, weights them differently [15]. Whereas absolute contraindications immediately discourage ECMO therapy, relative contraindications should trigger a very thorough consideration of this option.

Although relative contraindications should not per se exclude patients from a life-saving procedure such as ECMO, their concurrence may lead to the decision to forgo this procedure. When relative contraindications add up, they might accumulate to a point where they (should) be considered absolute contraindications.

The core question to be answered is as follows: Will the patient indicated for ECMO treatment really benefit from this treatment or will it predominantly place an additional burden on them? For this often challenging and emotionally stressful decision, it is necessary to weigh relative contraindications against indication in the individual patient, taking into account risks and complications. This decision-making process is complex and far from able to be worked through using a simple checklist. Below, we try to clarify this process.

Once it has become clear that extracorporeal treatment is indicated on the basis of gas exchange and/or lung mechanics alone, a search for contraindications must begin immediately. This process can be assisted by a referring center, being aware of potential contraindications, but ultimately must be performed by the (potentially) receiving ARDS/ECMO center. Each identified contraindication must be individually assessed for its severity and extent. For example, one contraindication may be weighted low in the individual assessment (e.g., bacterial pneumonia with known pathogen and targeted treatment for 2 days in immunosuppression due to renal transplantation, see Section 5.2), whereas another might be assessed as more severe (multiple organ failure, i.e., SOFA score 14). However, the more contraindications are present, the more difficult it becomes to reconcile the individual weighted factors. Ultimately, all factors present need to be considered and a decision made that is as evidence-based as possible, but ultimately represents an educated guess about the expected benefit of ECMO use in an individual case. This decision is likely to be more nuanced when made by a team that includes the most experienced ECMO caregivers.

To assist with this process, we reviewed the current literature of actual and former contraindications for the initiation of extracorporeal membrane oxygenation; contraindications for the continuation or indications for withdrawal can be derived from our work presented here but are explicitly not the focus of this article.

## 2. Methods

We searched the Medline database (via PubMED) combining the following MeSH (Medical Subject Headings) terms: “vvECMO”, “contraindication”, “contraindicated”, “not indicated”, “refusal”, “do not resuscitate”, “DN(A)R”, “cancer”, “advanced cancer”, “malignancy(-ies)”, “ICH”, “intracerebral hemorrhage”, “intracranial bleeding”, “cerebral herniation”, “intracranial hypertension”, “ICP”, “(increased) intracranial pressure”, “TBI”, “traumatic brain injury”, “brain dead”, “diffuse axonal injury”, “trauma”, “severe trauma”, “polytrauma”, “multiple trauma”, “pulmonary fibrosis”, “(lung) transplantation”, “hematologic malignancy(-ies)”, “HSCT”, “human stem-cell transplantation”, “BMT”, “bone-marrow transplantation”, “GvHD”, “graft-versus-host disease”, “immunocompromised”, “immunosuppression”, “advanced age”, “old”, “elderly”, “very old”, “time on ventilator”, “right-heart failure”, “pulmonary hypertension”, “SAPS II”, “SOFA score”, “PRESERVE score”, “RESP score”, “PRESERVE score”, “Jehovah’s Witness”, “no anticoagulation”, “obesity”, “obese”, “long run”, “runtime”, “time on ECMO”, and “COVID-19”. We limited the search to original articles, case reports/series, meta-analyses, and systematic reviews in adult humans that were available in English or German, with no limitation regarding the date of publication. The first search with the aforementioned criteria revealed 1204 articles in total; after removal of duplicates, 477 were left. Abstracts of all 477 articles were individually checked for eligibility; during this process, 372 articles were additionally discarded. Lastly, the selected articles were scanned for references from relevant studies with the same selection criteria as aforementioned which unveiled an additional 17 articles. The two investigators (L.-O.H. and O.M.) reviewed the identified abstracts independently and agreed on the final selection of 105 articles for inclusion; full texts were available for all studies finally selected. The study was carried out according to the Preferred Reporting Items for Systematic Review and Meta-Analyses (PRISMA) guideline.

We extracted data regarding survival status and the respective outcome; cumulative data are reported where available.

Statistical analysis was conducted for outcome prediction scores with respect to differences between outcome groups using Fisher’s exact test. Odds ratios were calculated from the publications where possible. For statistical analysis, we used SPSS (International Business Machines Corporated (IBM), Armonk, NY, USA, Version 26.0); statistical significance was assumed at *p* < 0.05.

## 3. Indications for the Initiation of ECMO

Although contraindications to the use of ECMO are largely relative and increasingly questioned, indications for the use of ECMO are widely agreed upon.

Currently almost universally used as rescue-therapy, evidence is accumulating that ECMO treatment could be even more beneficial in ARDS if instituted early, i.e., before the injurious effects of conservative treatment (i.e., ventilator induced lung-injury (VILI) or patient self-inflicted lung injury (P-SILI)) develop [16].

**Figure 1:**
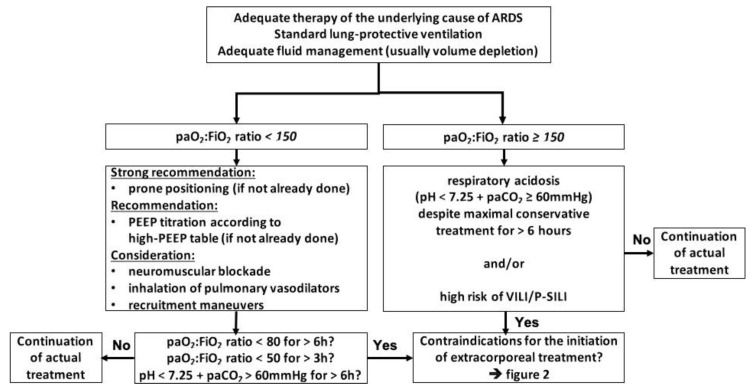
Algorithm to indicate ECMO initiation; adapted from [19], modified from [1,5,20]. Abbreviations: ARDS = acute respiratory distress syndrome, paO_2_ = partial arterial pressure of oxygen, FiO_2_ = inspiratory oxygen fraction, paCO_2_ = partial arterial pressure of carbon dioxide, PEEP = positive end-expiratory pressure, VILI = ventilator-induced lung injury, P-SILI = patient self-inflicted lung injury.

Currently, ECMO is most likely to be initiated by the majority of centers only if the following basic measures have been performed: the underlying cause of ARDS is identified and adequately treated, the patient is adequately resuscitated, which in most cases of ARDS means volume depleted, and the use of lung-protective ventilation strategies was implemented. If the paO_2_:FiO_2_ ratio remains below 150, prone positioning and ideally personalized adjustment of PEEP toward higher levels [17] should be established. If these measures and possibly less beneficial rescue therapies such as neuromuscular blockade or NO inhalation [18] were used and the paO_2_:FiO_2_ ratio remains below 80 for 6 h, below 50 for 3 h, respectively, the initiation of ECMO is indicated. The same applies for respiratory acidosis with a pH less than 7.25 sustained for more than 6 h despite maximal conservative treatment regardless of the paO_2_:FiO_2_ ratio [19] (Figure 1).

Although the rule to exhaust conservative strategies before considering ECMO is not always consistently followed, and the algorithms and decision trees used to determine the failure of these measures vary, they are generally well accepted.

## 4. Absolute Contraindications to the Initiation of ECMO

### 4.1. Refusal of the Use of Extracorporeal Techniques by the Patient

Refusal of possible therapy is a fundamental right of a person of sound mind. It can be expressed in the form of stock phrases in an advance directive, an actually expressed patient will, or a presumed/actual patient will by relatives/legal representatives in the case of a currently incapacitated person. The refusal of a possibly life-saving therapy must be respected if the person is aware of the implications of the decision to refuse treatment. However, caution must be exercised when this refusal is expressed by the next of kin/legal representatives. If there is even the slightest doubt about the legitimacy of this refusal, it is best to err on the side of therapy, with the option of discontinuing any therapy initiated at an early stage [21].

### 4.2. Advanced Stage of Cancer

No studies were identified that explicitly reported the effect of vvECMO on outcome in advanced cancer stages; thus, we attempted to make an analogy.

The principle “primum nil nocere” has been handed down from the beginnings of medicine; this paradigm is still relevant, perhaps even more so in modern intensive care medicine with its seemingly infinite measures and treatment options. It is the responsibility of the bedside physician not to initiate treatment that is unlikely to succeed in the broadest sense. The reduced life expectancy due to an advanced stage of cancer represents one of those circumstances in which ethical and medical triage can confluence. Although life expectancy cannot be reliably predicted today, advanced stages of cancer are generally associated with a reduced life expectancy, and treatments to prolong life in these stages are usually rejected. However, ECMO has been reported to facilitate palliative interventions [22,23,24,25].

Treatment goals in these patients require a very clear definition. Apart from a fundamental discussion of economic burden and distributive justice, initiating ECMO therapy for a limited period of time, to achieve a prespecified treatment goal, can be justified for individual patients.

Individual net gain for a patient burdened with end-stage cancer needs a thorough evaluation. We believe that patients facing a life expectancy of less than 5 months should not spend them in an ICU supported by an ECMO but rather in a palliative setting with symptom control close to family and friends. It is important to note, however, that assessment and attitudes of patients, relatives, and healthcare providers vary in this regard.

### 4.3. Fatal Intracerebral Hemorrhage/Cerebral Herniation/Intractable Intracranial Hypertension

Supratentorial mass lesions due to various reasons (e.g., intracranial hemorrhage (ICH), tumors, and cerebral edema as a consequence of traumatic brain injury (TBI)) can result in fatal transtentorial herniation. Rapid expansion of a supratentorial mass displaces the temporal lobe medially, after which the Uncus herniates over the edge of the tentorium cerebri; further progression leads to impaction of the temporal lobe in the tentorial notch, resulting in fatal secondary ischemia of the brainstem [26]. To disrupt this progression, medical and surgical measures have been advocated for decades. In Table 1, the literature from the last 3 decades is summarized with regard to mortality, as well as functional outcome of intracranial hypertensive conditions. There is a paucity of literature on ECMO in these populations [22], therefore, we tried to establish an analogy. 

We found that mortality was relatively low depending on the primary pathology. However, more than 50% of survivors remain in a low to very low or even vegetative functional state (Table 1).

According to data presented in Table 1, we strongly advocate *against* initiation of ECMO for acute respiratory failure in cases of intractable/uncontrollable intracerebral hypertension and/or cerebral herniation, let alone fatal intracerebral hemorrhage. 

### 4.4. Irreversible Destruction of the Lung Parenchyma without the Option of Transplantation

No studies reporting explicitly on the effect of vvECMO on this topic were available; therefore, we reviewed the literature on pulmonary fibrosis and present our assessment and experiences.

To effectively exchange gas, the lung parenchyma must be very delicate, making it very efficient but vulnerable at the same time. The mechanism that damages lung parenchyma is usually an inflammatory process either directly due to acute infection or secondary due to a chronic process such as inhalation of tobacco smoke or air pollution. The response to an injurious event depends on the type, intensity, and number of damaged cells. Following acute but limited superficial damage (e.g., airborne infection, irritants, airborne toxins) the epithelial lining of the respiratory tract can stimulate an effective regenerative cascade emanating from adjacent healthy epithelium [47]. If, however, the tissue structure/structural integrity of the lung provided by the basal membrane that underlies the alveolar epithelium is damaged, tissue is repaired rather than regenerated. Repairing in this context results in replacement of the normal/functional tissue architecture with fibrous tissue, consistent with scarring [48]. The same process is obviously true for mechanical damage (i.e., disruption) to lung tissue due to direct impact. Idiopathic pulmonary fibrosis is another entity that leads to irreversible destruction of lung parenchyma [49]. Depending on the extent of scarring/fibrotic transformation of the lung parenchyma, gas exchange is affected to various degrees. Fibrotic scar tissue will not contribute to gas exchange and will not heal but progress. Therefore, impaired gas exchange due to fibrotic tissue transformation in conjunction with contraindications to lung transplantation (see Section 4.5) represents an absolute contraindication to the initiation of vvECMO. However, there are situations where extensive pulmonary fibrosis is present, but gas exchange is not severely altered and patients do not experience severe restrictions. If this satisfactory lung function at baseline is worsened by acute infection, this situation could/should represent a relative contraindication in individual cases [50,51]. Unfortunately, no guidance is available on the extent of fibrosis still “acceptable” to initiate ECMO treatment, while the mode of rating the extent of fibrosis remains ambiguous. 

Regular computed tomography of the lungs does not contribute fundamentally to the assessment of the extent and severity of fibrosis, even more so if it is not performed in inspiratory hold.

Histological evaluation of the lung parenchyma requires samples that, in order to be meaningful, are often difficult to obtain and inherently only represent a small section of the organ. In addition, the procedure of obtaining a specimen usually by forceps biopsy during a bronchoscopy puts the patient at risk of bleeding and a period of worsening gas exchange. Above all this, the natural course, as well as the outcome, is unpredictable, and disease activity is usually monitored clinically [52].

Based on experience of the authors, the morphological extent of fibrosis is less important than the clinical baseline status of the patient before the onset of the acute situation; this is consistent with the current literature [52]. Taking into account that pulmonary status can only reach the baseline level in the best cases, in our opinion, baseline pulmonary functional status is the single most important factor predicting patient-centered outcomes and should not be ignored in favor of an image impression.

### 4.5. Contraindications to Transplantation without the Option of Sufficient Lung Healing

Survival rates after lung transplantation have significantly improved in recent years, mainly due to improvements in donor selection, organ preservation, and management of postoperative complications [53,54]. However, in the context of respiratory failure with an ambiguous chance of sufficient lung healing to be removed from extracorporeal support and the existence of a contraindication (Table 2) [55], vvECMO should be avoided. 

## 5. Relative Contraindications to the Initiation of ECMO

### 5.1. Advanced Age >70 Years

Age is a well-established risk factor for mortality in the ICU [56,57]. Due to an increasing proportion of old and very old people and an increasing life expectancy, more and more old and very old patients are admitted to an ICU with acute respiratory failure, potentially requiring extracorporeal life support [58]. The outcome of elderly patients treated with ECMO support has been evaluated in several trials, all showing moderate to high mortality of 50% or more in patients 65 years and older (Table 3). On the contrary, single case reports describe good survival and outcomes in patients 70 years and older [59,60]. Personal experience of the authors confirms both. We consider a mortality rate of 50–60% to not be an absolute contraindication for initiation of ECMO, particularly because different causes of ARDS vary in their mortality rates and should be taken into account. However, starting above 70 years, age should be seen as a relative contraindication. This could still be acceptable if the patient is otherwise in reasonable condition. However, if combined with an increasing number of relative contraindications such as those in the sections below, it should more and more be regarded an absolute contraindication in accumulation.

### 5.2. Immunocompromized Patients/Pharmacological Immunosuppression

Immunosuppression is an increasing phenomenon due to medical treatments (high-dose and/or long-term steroid treatment, immunocompromising drugs, and chemotherapy), solid organ transplants, or primary immune deficiency. Owing to extended indications and early, aggressive treatments, an increasing number of patients is expected to live for many years in an immunocompromised state, which puts them at risk for severe infections. Pulmonary infections with bacteria, viruses, fungi, and even parasites are common and the leading cause for intensive care admission, often in the shade of acute respiratory failure. Infections in immunocompromised patients are associated with an increased mortality, especially if respiratory failure and the need for mechanical ventilation ensue [63,64]. Knowledge of the mechanism of immunosuppression in conjunction with the most likely pathogen should lead to an early and aggressive treatment of the anticipated pathogen to get the situation rapidly under control. However, if respiratory failure is severe, the question of ECMO treatment arises. Table 4 summarizes the available studies that examined vvECMO in immunosuppressed patients. In all studies, mortality ranged up to 70%. Outcome data of specific immunosuppressed states are mostly missing.

According to available evidence, we advocate to rate immunosuppression as a relative contraindication to the initiation of vvECMO with the following consideration: if a pathogen has been identified and specific treatment is available and (about to be) started, a time-limited trial of ECMO treatment seems justified. If no pathogen has been identified or no specific treatment is available, regardless of the reasons, we advocate against the initiation of extracorporeal treatment. This statement refers only to the immunosuppressed state itself without having taken the rest of the patient’s state into consideration, which may modify the decision either way.

### 5.3. Time on Injurious Ventilator Settings >7 Days

Extracorporeal life support by vvECMO is currently viewed as a last-resort treatment if extended (“rescue”) conservative measures have failed to stabilize/improve respiratory status/gas exchange [2]. However, the days spent on a ventilator prior to ECMO initiation have been shown to be an important independent factor of mortality (Table 5). From the literature summarized in Table 5, one can deduce that, from a time of 5 days on a ventilator, mortality starts to increase. However, only extended periods, probably 7 days or more of obviously injurious ventilation, if at all, should be considered a relative contraindication to the initiation of ECMO. Data on the effect of lung protective ventilation during the ventilator period prior to ECMO treatment are not available. In all studies so far, injurious ventilation, for example, delta pressure >15 cmH_2_O and/or inspiratory pressure >35 cmH_2_O, was applied in both groups! Notably, the actual ELSO guidelines state that “many centers do not consider time on ventilation a contraindication” [68]. This is in accordance with the authors’ practice at their center. 

When requesting ECMO, information about the duration of (injurious) ventilation should be obtained. ECMO should be considered even in long-standing ventilation with only a short period on injurious settings or, rather, especially in these cases due to the acuity of the situation. Due to its ease of calculation, its broad availability, and close context with the mechanical properties of the lung, we actually regard the delta pressure as the most reliable marker of injurious ventilation that is widely available. Mechanical power is also a good marker for estimating the extent of injurious ventilation. However, it is undisputed that long periods of unfavorable ventilator settings in conjunction with other relative contraindications will often result in refraining from ECMO initiation.

### 5.4. Right-Heart Failure

No studies reporting explicitly on the effect of vvECMO on outcome in right-heart failure were available; therefore, we establish an analogy below on the basis of basic physiology. 

In ARDS, right-ventricular dysfunction is common [92,93,94]. It develops not only from pulmonary hypertension caused by the underlying pathophysiology itself [95], but also from hypoxia and hypercapnia [96,97,98], as well as mechanical ventilation [99,100,101]. However, the effect of pulmonary hypertension in ARDS on mortality is not unequivocally established [94,99,102,103,104]. Mechanistically, right-ventricular dysfunction might appear as an absolute contraindication for vvECMO, since the oxygenated blood from the ECMO will need to be pumped past the pulmonary circulation into the left ventricle in this setting. Recently, convincing beneficial effects on right-ventricular function due to significant decreases in mean pulmonary artery pressure after initiation of vvECMO have been reported [105]. These effects are based on a reduction in hypoxic pulmonary vasoconstriction, as well as decreases in paCO_2_ [74,106]. Thus, instead of starting vaECMO right away, which is associated with more complications than vvECMO [107,108], an approach of a trial of vvECMO and, in cases of failure, upgrading to vavECMO has been advocated [109] and seems reasonable.

### 5.5. Hematologic Malignancies, Especially Bone Marrow Transplantation and Graft-Versus-Host Disease

Advances in hematologic malignancy therapy (new chemotherapeutic agents and hematologic stem-cell transplantation (HSCT)) have improved patient outcome, and it is becoming more common for these patients to require admission to the ICU due to life-threatening conditions, [110], mainly acute respiratory failure (ARF) [111]. Admission due to ARF is associated with poor outcomes [112,113,114] and worsens if invasive mechanical ventilation is needed [115,116]. Nevertheless, efforts have been made in patients with hematologic malignancy to bridge ARF to recovery by use of ECMO [76,80,117,118,119,120,121,122]. All these trials conducted over a period of more than 10 years consistently demonstrated a high ICU and in-hospital mortality of more than 50% for patients treated with ECMO (Table 6). Moreover, extremely high mortality rates of patients that developed ARF after hematologic stem-cell transplantation or graft-versus-host disease (GvHD) of 100% have been reported in four trials; two additional trials reported an in-hospital mortality of two-thirds of HSCT patients treated with ECMO. 

While the prognosis for patients early after HSCT is grim, patients who acquire refractory ARDS later after HSCT may be eligible again for ECMO [119]. Immune reconstitution is generally reacquired after 6 months, and chances of survival are probably increased by then [123]. Therefore, the current literature suggests viewing the early phase after HSCT as an absolute contraindication to ECMO treatment. In turn, if HSCT has been completed some time ago, probably when immunosuppressive medication is tapered/immunocompetence is regained [123], it should become a relative contraindication. Along these lines, at this time, GvHD is also very uncommon [119].

**Table 6:** Evidence for the use of vvECMO in hematologic malignancies and the respective outcome.

Study	ICU Mortality	Hospital Mortality	Bone Marrow Transplant/HSCT Mortality (Hospital)
Gow et al. 2010 [117]	61%	68%	50%
Wohlfarth et al. 2014 [76]	50%	50%	100%
Kang et al. 2015 [118]	100%	100%	100%
Choi et al. 2016 [80]	n/a	80.9%	n/a
Wohlfarth et al. 2017 [119]	n/a	81%	100% (GvHD)
Stecher et al. 2018 [124]	n/a	80%	100%
Cho et al. 2019 [121]	66%	88%	66.7%
Park et al. 2021 [122]	n/a	86% (OR 42.25 (9.53, 187.22))	85.7% (OR 64)

### 5.6. SAPS II Score ≥ 60 Points

The revision of the Simplified Acute Physiology Score (SAPS II) was introduced in 1993. This score was developed and validated in a large cohort of medical and surgical patients from 137 ICUs in 12 countries with the goal of providing a relatively simple and easy-to-collect and -calculate score that would estimate the risk of death on admission to the ICU, regardless of the exact primary diagnosis [125]. Since its publication, this score has been used extensively to compare patient populations and trials regarding their disease severity, especially in research, but also in clinical practice. Due to the timing of the development and validation of the SAPS II score in relation to the implementation of ECMO, this score has not been specifically validated in patients with ARDS/ECMO. However, in many trials involving patients on ECMO, the SAPS II score has been routinely reported as a measure of the severity, describing the investigated cohort. SAPS II has also been explicitly investigated with respect to the prediction of outcomes in patients on ECMO [83,91,126,127,128]. All of these trials found only moderate precision in predicting mortality in patients on ECMO. One trial concluded that low mortality can be expected with a SAPS II score of less than 80 points [126]. However, a SAPS II score of 80 points translates into a predicted mortality of more than 90%, which seems too liberal for a procedure such as ECMO. Other trials found the SAPS II score to not be helpful in outcome prediction of ECMO patients [127]. Two articles in this Special Issue even congruently discourage the use of the SAPS II score to base the outcome prediction or the decision to initiate or refrain from extracorporeal treatment [91,128]. From the aggregation of the literature presented in Table S1, no statistically significant difference between the groups of survivors and non-survivors can be found (*p* = 1.0). Nonetheless, non-survivors had a mean SAPS II score above 50, whereas survivors had a score clearly below 60 (46.89); this was confirmed in a study by Lee et al., who found a cutoff value of 58 [83]. Therefore, we advocate a SAPS II score of 60 points or more (which translates into a predicted mortality of around 75% or more) within the last 24 h before considering extracorporeal treatment to be a relative contraindication to its initiation.

### 5.7. SOFA Score >12 Points (mSOFA Score >8 Points)

The Sepsis-Related/Sequential Organ Failure Assessment Score (SOFA) was created in 1996 as a result of an initiative of the European Society of Intensive Care Medicine “to quantitatively and objectively describe the degree of organ dysfunction/failure over time in groups of patients or even in individual patients” [129]. Although, as already stated in its first description, “it is important to realize that the SOFA score is designed not to predict outcome but to describe a sequence of complications in the critically ill”, it has increasingly been used to predict mortality in various diseases/conditions [130,131,132,133,134,135]. All of these trials only found a fair precision in predicting mortality [82], which obviously varies across different groups of patients. Reliable studies in vvECMO patients are largely missing. 

In a mixed population of critically ill patients, a clear cutoff value of 12 points was established with respect to mortality; here, mortality jumps from around 50% to more than 80% [129]. From our review of the literature, we can confirm this cutoff point of 12 points; the difference we found between the survivor and non-survivor groups was statistically significant (*p* = 0.004) (Table S2). A SOFA score >12 points obviously indicates a multiorgan dysfunction syndrome (MODS) severe enough to exponentially increase mortality. Therefore, a SOFA score of greater than 12 points, regardless of whether it was measured on admission, as the highest value, or as the value at the time of indication to ECMO treatment, can strongly be advised to be a relative contraindication to its initiation.

Calculating the SOFA score in critically ill patients regularly faces inaccuracies because evaluation of the neurological status as assessed by the Glasgow Coma Scale (GCS) is very difficult in sedated patients and frequently overscores the neurological component. To account for this inaccuracy, a modified SOFA score (mSOFA) has been proposed [136]. This modified score rates the neurological component by scoring the patient with the best assumed score without sedation (i.e., 15 if no history of neurological disorder is present). In all the studies referenced in Table S2, the regular SOFA score was used, and this was also initially the case for this review. However, if mSOFA prevailed or was used regularly in some centers, we calculated the mSOFA score from the publications and included it in Table S2. The cutoff value when the mSOFA score becomes a relative contraindication is greater than 8 points.

### 5.8. PRESERVE Score ≥ 5 Points

In the process of searching for a specific model to predict outcome of ARDS patients and to aid in the decision to initiate ECMO, Schmidt et al. conducted a study “to identify factors associated with death by 6 months post ICU discharge for ARDS patients treated with the latest generation ECMO systems and to assess long-term survivors’ health-related quality of live (HRQL) and psycho-emotional sequelae” [137]. In their analysis of 140 patients from three experienced ARDS/ECMO centers in France, they identified eight parameters that were independently associated with death by 6 months post ICU discharge in a multivariable analysis: age, body mass index (BMI), immunocompromised status, SAPS II, days of mechanical ventilation, no prone position before ECMO, positive end expiratory pressure (PEEP), and plateau pressure. Of these factors (with the exception that SAPS II was replaced by SOFA score), the PRESERVE (Predicting Death of Severe ARDS on vvECMO) score was derived by assigning weighted points to each parameter (age was split in three categories) and summing them to build the final score of 0 to 14 points.

From the Kaplan–Meier estimates of the derivation trial, the highest mortality rate was seen in the group of patients with a PRESERVE score ≥7 points, whereas mortality rates greater 50% can be found in patients with ≥5 points [137]. Although different percentages have been reported, a trend that patients with a PRESERVE score ≥5 points experience high mortality rates of around and above 50% seems common [78,83,91,128,138] (Table S3). However, discrimination between survivors and non-survivors is only moderate (ROC-AUC around 0.6) for most trials. Therefore, using the PRESERVE score as the only aid for decision to initiate ECMO is generally discouraged. We propose—due to its moderate discrimination and ease of calculation—to collect this score in every patient fulfilling ECMO initiation criteria and use it as an additional source to contribute to the decision-making process.

### 5.9. RESP Score Worse Than −2 Points

From the same researchers that also constructed the PRESERVE score, another survival prediction score was proposed only 1 year later: the Respiratory Extracorporeal Membrane Oxygenation Survival Prediction (RESP) score. This score was constructed retrospectively from the ELSO database (Extracorporeal Life Support Organization) using logistic regression and bootstrapping [139]. The score derived from this method can range from values of −22 to 15, breaking down into five risk categories (I–V). Of these, risk category IV (−2 to −5 points) already translates into a mortality rate of 67%, as has consistently been shown [78,83,128,138,139]; in our summary of the literature, even higher/less negative scores were found in non-survivors (Table S4).

The RESP score also has a moderate discrimination between survivors and non-survivors, albeit slightly better than the PRESERVE score (ROC-AUC around 0.7–0.75). Similar to PRESERVE, using this score as the only aid to decide whether to initiate ECMO or not is discouraged, but it can be used as an additional resource.

### 5.10. PRESET Score ≥ 6 Points

The newest of the outcome prediction scores for the initiation of ECMO therapy is the Prediction of Survival on ECMO Therapy (PRESET) score [86]. It provides some advantages over the other risk scores mentioned before. First, it consists of only five items that are captured during daily routine (mean arterial pressure, lactate concentration, arterial pH, platelet concentration, and hospital days before ECMO). The items in each category are weighted by assigning individual points (0 to 5), which are summed to result in a final score of 0 to 15 points. The summed score allows a breakdown into three distinct risk categories that consist of 5 points each. These risk categories finally translate into a mortality prediction (category I 26% mortality, category II 68% mortality, category III 93% mortality). Interestingly, the derivation of this score only identified extrapulmonary factors that predict mortality. This fact is unexpected in light of the other prediction scores mentioned, the underlying severe lung pathology, and the known problems of ventilating these patients adequately without increasing damage to their lungs. However, this score proved to be a good predictive value in COVID-19 patients [140], which is advantageous in light of the current pandemic. Moreover, a recent evaluation of this score revealed that it performed best amongst the other ECMO prediction scores; however, with an AUC of 0.658, the absolute performance was still only moderate. Authors of recent evaluations of ECMO prediction scores discourage the use of any of the available scores as a single decision tool [83,128]; however, acting with caution, the PRESET score could still be advantageous [141].

### 5.11. “Do Not Attempt Resuscitation Order” (DN(A)R Status)

The existence of “do not resuscitate” orders has increased with time [142], although a wide range of prevalence has been reported [143,144,145]. Only very few guidelines on writing DNR orders—the “when and how”—are available, e.g., by professional medical societies [21]. This wide lack of consensus among healthcare professionals has serious implications. DNR orders are frequently written/executed without family involvement, let alone patient involvement [15,144]! Consequently, in the same investigation, DNR orders were often found to be inappropriate with regard to the pre-arrest morbidity [144]. For most patients, family members, and healthcare professionals not involved in critical care medicine, DNR orders often refer to “classical” cardiopulmonary resuscitation (i.e., closed chest massage). However, in the intensive care unit, there are a wide range of “subtler” resuscitative measures (e.g., vasoactive medications, dialysis, ECMO, etc.) available that are usually not considered when these orders are documented. As a result, hospitals and individual physicians vary greatly in their use of life-sustaining treatments in patients with DNR orders [146,147,148,149]. No studies reporting explicitly on DN(A)R orders and vvECMO are available.

We recommend refraining from the assumption that every DNR order automatically means refusal of vvECMO therapy (for vaECMO, the situation could be more obvious), especially in situations where vvECMO is intended as a bridge to recovery or bridge to transplant. In cases of uncertainty, we support a treatment trial with a narrow timeframe. Therefore, we list DNR orders as a relative contraindication to vvECMO, unless this therapy is explicitly excluded in the order, which is consistent with a refusal for ECMO by the patient (see Section 4.1).

## 6. Factors Excluded as Contraindications/Additional Contraindications Only

### 6.1. Jehovah’s Witness/Refusal for Blood Transfusions

Since alterations of hemostasis (bleeding and thrombosis) are common complications of extracorporeal membrane oxygenation [150], the use of packed red blood cells (PRBC), as well as plasma, platelets, and blood components, is common during ECMO treatment. A recent meta-analysis concluded that, on average, 2.6 units of PRBCs are transfused per day on ECMO [151]. Therefore, the refusal for blood transfusions, most often encountered in patients of Jehovah’s Witness faith, could be counted as a contraindication for the use of ECMO. In recent years, case reports of Jehovah’s Witnesses safely treated without blood transfusion while on ECMO have been published [152,153,154,155]. Managing patients without blood transfusions while on an extracorporeal circuit is very rare; however, it is an important option for these patients and advocates even more for a center specialized and very experienced in ECMO treatment. The cited case reports demonstrate feasibility, and various interventions can aid in doing so. Therefore, the refusal of blood transfusions per se cannot exclude patients from ECMO treatment if indicated; rather, a very experienced center should be involved in caring for these patients.

### 6.2. Fixed Pupils/Missing Brainstem Reflexes in Acute Settings

Bilateral fixed pupils that do not react to light usually represent a strong indication toward a poor neurologic outcome up to brain death. However, similar to the famous saying “no one is dead until warm and dead”, reversible factors for fixed pupils such as drugs, poisonings, neuropathies, and other factors need to be excluded before fixed pupils and/or missing brainstem reflexes are regarded contraindication to ECMO. In a recent case series, fixed pupils were not a marker of poor neurologic outcome even after resuscitation [156]. We, therefore, suggest that fixed pupils and/or missing brainstem reflexes should be excluded as a finding when weighing contraindications to the initiation of ECMO; rather, further diagnostics should be instituted. If no other contraindications apply, ECMO should be started; if, however, other relative contraindications are present, fixed pupils should weigh them down.

### 6.3. Nonfatal Intracranial Hemorrhage/Restrictions on Therapeutic Anticoagulation

Traditionally, artificial circuits in which blood circulates outside the vascular system and back into the body require substantial anticoagulation to counteract the thrombogenic effect of the material from which tubes and membranes are made. However, in recent years, progress has been made in making these materials much more biosimilar so that they inhibit coagulation in a way that is close to the normal endothelium [10,11,12]. Moreover, coagulation happens way less in circuits that run with high flow rates such as ECMO circuits than in circuits that run with lower flow rates such as in renal replacement therapy. From data available, it might be tempting to use high-flow circuits with a reduced dose of anticoagulant.

Available literature on this topic is scarce at best; a recent paper described a before-and-after evaluation of changing anticoagulation practice from therapeutic to prophylactic dose. In this study, no differences were found in survival, bleeding, thrombotic complications, and transfusion requirements. The authors concluded that “vvECMO can be a safe option in patients with traditional contraindications to anticoagulation” [157]. The same results were presented in a systematic review [158]. However, in an even more recent retrospective analysis, it was found that “ECMO management with high-dose heparinization was associated with lower rates of oxygenator changes and thromboembolic events when compared to a low-dose heparinization strategy” [159].

Experienced ARDS/ECMO centers might also consider running a high-flow circuit completely without anticoagulation for a limited time [158,160,161,162,163,164,165,166]. Completely abstaining from anticoagulation should not be the standard of care; however, it enables ECMO treatment in patients with contraindications to anticoagulation such as intracranial hemorrhage. Therefore, the available literature avoids specifying contraindications to complete systemic anticoagulation as contraindications to ECMO treatment. If considered, this should be performed in a very experienced center with a care team immediately available to change a clotting circuit if necessary.

However, no large studies of sufficient quality are available that investigate the effects of different anticoagulation agents and targets in vvECMO. A review by Kato et al. found no differences between strategies [167]. Moreover, no randomized controlled trials on anticoagulated vs. anticoagulation-free vvECMO in intracranial hemorrhage are available.

### 6.4. Traumatic Bain Injury/Diffuse Axonal Injury

No studies reporting explicitly on the effect of vvECMO on outcome in traumatic brain injury/diffuse axonal injury were available; therefore, we attempt to establish an analogy. 

Traumatic brain injury is a leading cause of morbidity and mortality with an estimated 69 million individuals affected worldwide each year [168] and a reported mortality rate of 10.8/100,000 [169]. In addition to focal lesions resulting from direct impact (e.g., coup and contrecoup lesions), injuries that are largely concealed from regular brain imaging (computed tomography) are also common (diffuse axonal injury, DAI). This type of lesion is often the result of motor vehicle accidents, but can also occur in other instances, such as falls. The underlying mechanism involves inertial forces acting on the head and resulting in a rapid (<50 milliseconds) rotational acceleration of the brain as a result of unrestricted head movement that is sufficient to deform the white matter. These forces are commonly referred to as shear forces, which is why diffuse axonal injury is often also called *shear brain injury*. Due to the mass of the human brain, its parts “slip” against each other, which leads to tensile elongation that damages the axonal cytoskeleton and, in turn, induces a complex cascade of reactions, accumulating in axonal pathology [170]. The usual pattern of this type of injury is multilocal and is especially common in midline structures such as the splenium and brainstem. This distribution pattern explains why coma is the most common presentation of DAI. However, in contrast to coma induced by focal lesions (e.g., hematoma resulting in herniation and brainstem compression) which often develops over time, coma induced by DAI often results in an immediate and prolonged coma without detectable mass lesions [171]. Although coma is usually associated with a bad outcome, this is way different for DAI. Analyses of patients with evidence of DAI report a low mortality rate of 2–25% [172,173,174,175,176], as well as a good functional outcome [172,175,177,178,179,180] and quality of life [173,177]. The presence of typical DAI lesions and the anatomical classification of imaging studies do not predict survival or functional status, let alone quality of life [173,175]. However, conflicting results have been reported for almost all the aforementioned aspects [172,175,177,181,182].

Consequently, the outcome in the presence of DAI is highly variable, and its prediction is imprecise at best. Current evidence does not support rating traumatic brain injury, the existence of DAI-specific lesions on imaging, and even coma following trauma to the head as a contraindication to the initiation of ECMO therapy. Rather, supported by the literature mentioned, we strongly advocate in favor of ECMO treatment if a good quality of life might ultimately be achieved. We acknowledge, however, that no cumulative data from original research on traumatic brain injury and the use of vvECMO are available.

### 6.5. Use of Vasopressors

The use of vasopressors in the critical care setting is not a measure of action, but a reaction to symptoms of circulatory depression. Furthermore, its dose is usually a measure of severity of circulatory depression. Therefore, the use of vasopressors should be viewed as an expression of organ dysfunction and its severity, which is incorporated into severity scores such as the SOFA score [129]. Therefore, the fact that vasopressors are required in an individual patient, as well as the dose needed, is a possible marker of organ failure and could be viewed as a relative contraindication to the initiation of ECMO treatment. Au contraire, buildup of CO_2_ with consecutive respiratory acidosis is an established indication for ECMO treatment (Figure 1). This acidosis negatively impacts the efficiency of vasopressors, necessitating a dose increase, which in turn impairs microcirculation, leading to increased lactate levels, which again add to acidosis and so on.

No differences in vasopressors were found between survivor and non-survivor groups in our review of the literature (Table 7). Of the 13 studies that reported the proportion of patients who needed vasopressors before the initiation of ECMO, only three (all reporting hematologic patients) found a significant difference [110,111,112], while the other 10 studies did not report a difference in survival with respect to the use of vasopressors. One retrospective analysis focusing on the use of vv vs. vaECMO with regard to vasopressor and/or inotropic use pre ECMO found that, in patients with two or more vasopressors/inotropic agents, the use of vv compared to vaECMO was even associated with increased survival [87]. These data indicate strongly that the use of vasopressors at the time of ECMO indication cannot be considered a contraindication, regardless of the dose.

### 6.6. Obesity

Obesity has been associated with an increased risk of all-cause mortality [185] and identified as an independent risk factor for death in patients treated in intensive care units [186]. Regarding respiratory failure, it was found that, during the H1N1 pandemic, obesity was a risk factor for hospitalization and even death due to influenza pneumonia [187]. However, in a post hoc analysis of a randomized controlled trial, no difference in adjusted hospital mortality across BMI strata in moderate to severe ARDS has been reported [188]. Two recent studies reported the same results of no difference in BMI between survivors and non-survivors [83,128].

Furthermore, from our literature review, we found that, in patients treated with ECMO for severe ARDS, mortality decreased, while, in many trials, BMI increased (Table 8). This finding is named the “obesity paradox”. Although theories exist to explain this finding, none of them have been proven so far. In the PRESERVE score, a BMI >30 kg/m^2^ is weighed negatively, which corresponds to a “protective condition” [137]. 

From our reading of the condensed literature in Table 8, all published after the PRESERVE score was proposed, we cannot argue in favor of a protective effect of (morbid) obesity. However, it seems that obesity should not be considered a contraindication to the initiation of ECMO.

### 6.7. Trauma/Polytrauma

In patients with severe traumatic, thoracic injury, the lung fails in about 20% of cases [198]. Acute respiratory failure can be caused by the primary insult (penetrating or blunt chest trauma), resulting in pulmonary hemorrhage, hematothorax and pneumothorax, lung contusion, or respiratory instability caused by bone fractures and flail chest. Furthermore, patients with lung contusions are at increased risk of a second hit by pneumonia and the ventilation-induced inflammatory response [199].

Since severe trauma often presents in conjunction with coagulopathy and consecutive bleeding, and since the ECMO circuit itself can also lead to coagulopathy and bleeding, trauma has been considered a contraindication to its use [158,195].

Recent data suggest ECMO as a rescue measure in these patients. An analysis of the ELSO registry revealed a survival rate to discharge of 61% in trauma patients [200]. Extended advanced monitoring is suggested to detect early deterioration, ideally as a baseline before initiation of ECMO to prevent potentially deleterious bleeding or thromboembolic complications [201,202]. Using extracorporeal lung assist has been reported in various circuit compositions, with all of these reports describing feasibility without an increased risk of bleeding and survival benefits, as well as high survival rates [161,162,203,204,205,206,207,208,209,210,211,212,213,214,215,216,217]. Some authors even reported using it in severe bleeding situations [158,216,217,218]. To gain the greatest benefit, a decision to initiate ECMO should not be delayed [209,213,214,219]. In particular, the injury severity score (ISS) did not show an influence on survival for patients treated with or without ECMO [203,204,206,207,210,211,212]. Therefore, ECMO should be initiated early in trauma patients if indicated, rather than considered a contraindication (Table 9).

### 6.8. Return of Spontaneous Circulation (ROSC) after Cardiac Arrest Due to Hypoxemia/Hypercarbia

Patients in cardiac arrest and patients with severe impairment of cardiac function after return of spontaneous circulation are usually considered candidates for vaECMO. vaECMO is often suggested in these patients because cardiac causes such as myocardial ischemia or pulmonary embolism usually underlie the cardiac arrest. However, if severe hypoxemia due to respiratory failure is reason for the cardiac arrest, which has been found to contribute in up to 45% of cardiac arrests [222], vvECMO might very well be a consideration, as it reverses the underlying hypoxemia causally. Cardiac function is usually not compromised in these patients, and right-heart strain—if existent at all—can rapidly be reversed once carbon-dioxide levels are normalized, which is also accounted for by vvECMO.

Although this line of reasoning seems straightforward, neither recommendations nor a reasonable body of evidence exists to support this. On one hand, this is probably because vaECMO is frequently the modality of choice. On the other hand, providers might be reluctant to initiate vvECMO after cardiac arrest due to the assumption that the outcome will be poor.

In our review of the available literature, we found only two articles that explicitly address this issue. Bhardwaj et al. found in their retrospective analysis of 20 patients treated with vvECMO after ROSC a survival to hospital discharge of 57% [223]. Although only a case report of a single case, Lee et al. described a favorable outcome without neurologic sequelae even after prolonged resuscitation (45 min) for respiratory failure in a trauma patient finally treated with vaECMO [224].

Although available evidence is limited, it does not support the conclusion that ROSC after cardiac arrest due to hypoxemia or hypercarbia is considered a contraindication to initiate vvECMO. We suggest to closely look at the timing and adequacy of resuscitation rather than the fact that resuscitation had to be undertaken.

## 7. Factor Excluded for the Termination of ECMO

### ECMO Runtime

In an early single-center experience, Mols et al. reported a shorter ECMO runtime for survivors of ECMO treatment [70]. However, these findings could not be reproduced in more recent reports [121,127,225,226,227,228]. In a very recent analysis of the ELSO database, the authors specifically looked at prolonged ECMO runs >14 days and found survival rates of greater than 50% [89]. Therefore, from the available evidence, it can be presumed that ECMO runs of several weeks and, if applicable, oxygenator changes are justified if the potential for recovery of lung function is given and no additional severe organ failures exist simultaneously.

## 8. Special Consideration

### COVID-19

Respiratory disease caused by severe acute respiratory syndrome coronavirus 2 (SARS-CoV-2) is termed COVID-19. At the time of writing this article, we are in a pandemic with hope of its end in sight. Although COVID-19 is still a very new disease and little about it is fully understood, let alone proven by evidence, certain patterns have evolved.

Patients with COVID-19 seem to tolerate hypoxemia astonishingly well, a circumstance that has been uncouthly called “happy hypoxia”, which is psychologically and medically wrong. 

With regard to ECMO treatment, not only gas exchange needs to be factored into the decision to initiate and resume ECMO treatment, but also respiratory drive, which can be extremely actuated in these patients due to unknown reasons so far. Gas exchange may take much longer to restore than in “regular” ARDS not due to SARS-CoV-2, let alone influenza. Most COVID-19 cases take several weeks of ECMO runtime to allow the natural lung to regain sufficient gas exchange. When gas exchange has mainly recovered and ECMO weaning is attempted, the extreme respiratory drive that is often found in combination with a still affected lung can lead to self-inflicted lung injury (P-SILI) if not rigorously controlled [229,230]. Our experience as a COVID-19 referral center is consistent with what has been described in the literature. COVID-19 warrants long ECMO runtime and, if necessary, several oxygenator changes or even recannulations, because these factors seem to not be associated with poor survival [91,184,227,231].

## 9. Discussion

Due to the increasing availability of extracorporeal lung assist devices and improvements in the biocompatibility of circuits, more and more physicians are considering ECMO.

However, ECMO treatment bears the risk of serious complications. These include the risk of mechanical damage from the large and stiff cannulas and bougies during implantation [232], bloodstream infections, bleeding due to coagulopathy, and possibly even irreversible organ damage to the liver, kidneys, and brain [233]. On the other hand, ECMO therapy can stabilize an acutely life-threatening situation and improve the outcome for many patients with severe, refractory acute respiratory failure. 

Ongoing ECMO therapy is usually seen as a contraindication to lung transplantation, at least in acute respiratory failure. Therefore, the lack of perspective in the case of inadequate recovery of lung function remains problematic. Consequently, the decision to provide ECMO treatment should be taken with great care. Availability and feasibility should not force initiation of ECMO. The decision must focus on the goal of achieving a meaningful, patient-centered outcome. To this end, treatment options should be clear and available, and contraindications and potential outcome options should be considered.

We identified factors that should immediately lead to avoidance of ECMO initiation. These absolute contraindications have been well established in clinical trials.

The situation is much more difficult with relative contraindications. Most of them, taken individually, will not negatively affect the outcome very much. Although the presented relative contraindications are not equally important, the simultaneous occurrence of more than three of these relative contraindications should be considered and evaluated as an absolute contraindication. The suggestion to count a patient with three relative contraindications still eligible for ECMO initiation is not based on evidence, but rather on the experience of the authors treating patients with ECMO for more than 15 years; it is a concept. 

**Figure 2:**
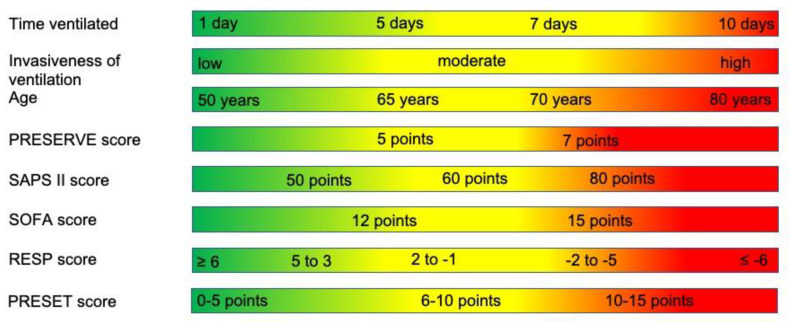
Sliding scales of relative contraindications. This figure is intended to illustrate the interplay between the continuous relative contraindications. A value in a red section in one or two categories can be countered by values in the green or at times yellow sections in another category.

Of the relative contraindications identified, some are categorical and, therefore, much clearer and easier to follow. Others are continuous, such as age and duration of ventilation; these variables must be evaluated dynamically. A young patient with immune suppression, a SOFA score of 14, and a duration of ventilation of 10 days with a delta pressure of 16 would probably not be considered for ECMO, whereas a 72 years old patient with type II diabetes and arterial hypertension who has been ventilated for 10 days with 6 cm H_2_O PEEP and 16 cm H_2_O peak pressure at a FiO_2_ of 0.5 and whose condition has acutely deteriorated would probably be cannulated. We have tried to visualize this situation in the form of sliding scales in Figure 2.

**Figure 3:**
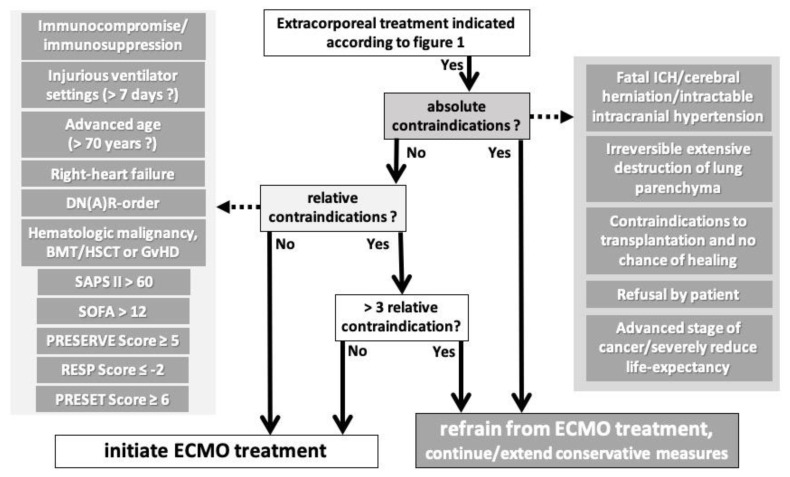
Algorithm to include contraindications in the process of initiation of ECMO treatment after the indication has been confirmed according to Figure 1. Abbreviations: DN(A)R = do not attempt resuscitation, SAPS II = Simplified Acute Physiology Score first revision, SOFA = Sepsis-Related/Sequential Organ Failure Assessment, PRESERVE = Predicting Death of Severe ARDS on vvECMO, RESP = Respiratory Extracorporeal Membrane Oxygenation Survival Prediction, PRESET = Prediction of Survival on ECMO Therapy, BMT = bone marrow transplantation, HSCT = human stem-cell transplantation, GvHD = graft-versus-host disease, ICH = intracranial hemorrhage, ECMO = extracorporeal membrane oxygenation.

We did not attempt to perform a statistical model to create another outcome prediction score for ECMO treatment. Rather, we attempted to create a relatively simple algorithm that can be easily followed by the care team, incorporating established outcome prediction scores (Figure 3). We are aware that some of the factors we have classified as relative contraindications are also part of the outcome prediction scores. We think that this fact still justifies counting these factors separately because they are common and well established. Nevertheless, we believe that three of these relative contraindications are easily and often found together, and that it is very liberal to still consider a patient with three relative contraindications for initiation of ECMO. Ultimately, it is a case-by-case decision of the caregivers (team). We hope that our compilation of the literature can be helpful as a basis for decision making.

Limitations: Although we conducted an extensive literature search, we cannot exclude the possibility that individual publications were missed. Many of the articles reporting outcome prediction scores were either not calibrated for the target population or not sufficiently powered to detect differences in relation to these scores. 

The cutoff point to rate a patient with three relative contraindications as still eligible for ECMO initiation is very conceptual in nature, purely based on the experience of the authors, and it can, therefore, be questioned; it needs a proof of concept. A retrospective analysis from the authors’ center is currently ongoing to provide supporting data.

## 10. Conclusions

To initiate extracorporeal lung support, it should not only be indicated but, more importantly, not be contraindicated. In addition to absolute contraindications, which should almost unquestionably lead to discontinuation of therapeutic consideration, relative contraindications must also be taken into account. Although the number is somewhat arbitrary and subject to controversy, more than three relative contraindications can probably accumulate to an “absolute contraindication”. We provided an easy-to-follow algorithm that incorporates absolute and relative contraindications to ECMO initiation and attempted to weigh all the pros and cons for an individual patient, helping the care team make the best-informed decision possible.

## Figures and Tables

**Table 1:** Outcome of different intracranial pathologies leading to intracranial hypertension. Abbreviations: GOS = Glasgow Outcome Scale, SDH = subdural hematoma, EDH = epidural hematoma, ICU = intensive care unit, TBI = traumatic brain injury, DC = decompressive craniectomy, ICH = intracranial hemorrhage.

**Study**	**Pathological Condition**	**Mortality**	**Worse Outcome** **(GOS ≤ 3 or Equal)**
Gurer et al. 2017 [24]	SDH/EDH	49.1% (ICU)	66.7% (6 months)
Gower et al. 1988 [27]	swelling after TBI	23% (ICU)	60% (≥ 2 years)
Gaab et al. 1990 [28]	swelling after TBI	14% (ICU)	22% (n/a)
Polin et al. 1997 [29]	swelling after TBI	23% (hospital)	63% (discharge)
De Luca et al. 2000 [30]	swelling after TBI	18% (n/a)	59% (n/a)
Taylor et al. 2001 [31]	swelling after TBI (children)	DC: 33% (1 week) medical: 42% (1 week)	46% (6 months)
Whitfield et al. 2001 [32]	swelling after TBI	23% (10 months)	31% (10 months)
Schneider et al. 2002 [33]	swelling after TBI	22.5% (6 months)	71% (6 months)
Albanèse et al. 2003 [34]	swelling after TBI	early DC: 52% (1 year) late DC: 23%	62% (1 year)
Aarabi et al. 2006 [35]	swelling after TBI	32.4% (30 days)	48.7% (30 days)
Wettervik et al. 2018 [36]	swelling after TBI	DC: 17% (6 months) Thiopental: 4% no specific treatment: 11%	DC: 60% (6 months) Thiopental: 48% no specific treatment: 27%
Sakai et al. 1998 [37]	cerebral infraction/malignant swelling	33% (2 months)	67% (2 months)
Qureshi et al. 2000 [38]	medical reversal of supratentorial masses	54% (hospital)	46% (Barthel & Rankin) (≥6 months)
Koenig et al. 2008 [39]	medical reversal of transtentorial herniation	67.6% (hospital)	77% (GOS 4 & 5)
Skoglund et al. 2005 [40]	transtentorial herniation after TBI	26% (≥6 months)	41% (≥6 months)
Kim et al. 2009 [41]	DC for TBI/ICH/infarction	TBI: 21.4% (6 months) ICH: 25% (6 months) Infarction: 60.9% (6 months)	TBI: 42.9% (6 months) ICH: 50% (6 months) Infarction: 69.6% (6 months)
Lan et al. 2020 [42]	DC for herniation after TBI	30.4% (6 months)	66% (6 months)
Delcourt et al. 2017 [43]	ICH	12% (90 days)	45.4% (90 days)
Chen et al. 2019 [44]	infratentorial ICH	8% (90 days)	28% (90 days)
Poon et al. 2014 [45] (metaanalysis)	ICH	46% (1 year)	up to 24% (1 year)
Pinho et al. 2019 [46] (metaanalysis)	ICH	36.3% (1 year)	n/a

**Table 2:** Contraindications to lung transplantation (adapted from Weill 2018 [55]).

Absolute Contraindications	Relative Contraindications
History of malignancy (<2–5 years disease free plus high risk of recurrence)	Age >65 years plus low physiological reserve
Significant dysfunction of another major organ system (heart, liver, kidney, brain)	Mechanical ventilation/extracorporeal life support
Uncorrected coronary artery disease	Controlled coronary artery disease
Unstable medical condition	Significant osteoporosis
Uncorrectable bleeding	Extensive prior chest surgery
Poorly controlled infection/resistant microbes	Colonization with resistant microbes
Inadequate social support	Infectious liver cirrhosis
Severe thorax deformity	HIV infection (unless treated adequately)
BMI ≥35 kg/m^2^	BMI 30–35 kg/m^2^
Nonadherence to medical therapy (recent & history)	Significant malnutrition
Inability to comply with therapy	Specific infections [55]
Active tuberculosis/contraindications to immunosuppression	Poorly controlled diabetes, hypertension, epilepsy, peptic ulcer disease, gastroesophageal reflux, or central venous obstruction
History of illicit substance abuse	
Inability to participate in rehabilitation	

**Table 3:** Evidence for the use of vvECMO in elderly patients and the respective outcome.

Study	Age Defining “Elderly”	No. of Patients Included Total	Hospital Mortality in the “Elderly”
Mendiratta et al. 2014 [58]	>65	368	59%
Karagiannidis et al. 2016 [8]	>80	1944	76%
Deatrick et al. 2020 [61]	>65 >55	182	83% 43%
Giani et al. 2021 [62]	>65	144	56%

**Table 4:** Evidence for the use of vvECMO in immunocompromised patients and the respective outcome.

Study	Disease State	ICU Mortality	Hospital Mortality	Odds Ratio
Cawcutt et al. 2014 [65]	HIV/AIDS	40%	60%	n/a
Schmidt et al. 2018 [66]	Mixed	66%	n/a	n/a
Huprikar et al. 2019 [67]	Acute leukemia	n/a	50%	n/a

**Table 5:** Evidence for the relevance of time on ventilator prior to ECMO and the respective outcome.

Study	Outcomes
Pranikoff et al. 1997 [69]	50% mortality after 5 days on ventilator (90% after 12 days)
Mols et al. 2000 [70]	No differences between groups
Hemmila et al. 2004 [71]	OR 1.20 (1.09, 1.31) (3.2 vs. 4.5 days) (OR 5.53 if > 8 days)
Beiderlinden et al. 2006 [72]	OR 1.064 (1.008, 1.123) (5.3 vs. 8.7 days)
Patroniti et al. 2011 [73]	OR 1.291 (29% increase each day)
Schmidt et al. 2013 [74]	*p* = 0.0008 between groups (3 vs. 7 days), OR 1.07
Enger et al. 2014 [75]	*p* = 0.013 between groups (2 vs. 5 days)
Mendiratta et al. 2014 [58]	*p* = 0.049 between groups (1.19 vs. 1.73 days)
Wohlfarth et al. 2014 [76]	*p* = 0.17 between groups (1 vs. 3 days)
Schmidt et al. 2015 [77]	OR 1.15 (1.06, 1.26) (2 vs. 4 days)
Klinzing et al. 2015 [78]	*p* = 0.14 between groups (1 vs. 4 days)
Cheng et al. 2016 [79]	*p* < 0.001 between groups (1 vs. 6 days), OR 4.71 (1.98, 11.23)
Choi et al. 2016 [80]	*p* = 0.11 between groups (4.5 vs. 4.77 days)
Huang et al. 2016 [81]	*p* = 0.093 between groups (0.5 vs. 1.8 days)
Hsin et al. 2016 [82]	*p* < 0.001 between groups (1 vs. 6 days)
Lee et al. 2016 [83]	*p* = 0.114 between groups (2.3 vs. 4.2 days)
Serpa Neto et al. 2016 [84]	*p* = 0.061 between groups (2 vs. 3 days)
Wu et al. 2016 [85]	*p* = 0.005 between groups (2.75 vs. 6.92 days)
Hilder et al. 2017 [86]	*p* = 0.140 between groups (1.08 vs. 1.67 days)
Kon et al. 2017 [87]	OR 0.998 (0.997–0.999), *p* = 0.001
Wu et al. 2017 [88]	*p* < 0.001 between groups (1 vs. 6 days)
Schmidt et al. 2018 [66]	*p* = 0.004 between groups (2 vs. 3 days)
Posluszny et al. 2020 [89]	*p* = 0.028 between groups (2.33 vs. 3.25 days)
Giraud et al. 2021 [90]	*p* = 0.01 between groups (3.79 vs. 8.67 days)
Supady et al. 2021 [91]	*p* = 0.006 between groups (3 vs. 6 days)

**Table 7:** Evidence of the lack of difference between survivors and non-survivors with respect to vasopressor use. Values are presented as percentage of patients for whom vasopressors were used in relation to the control group; one study presented absolute mean values. *p*-Values were extracted from the respective study.

Study	Vasopressors Survivors	Vasopressors Nonsurvivors	*p*-Value
Benoit et al. 2003 [111]	29.8%	70.2%	0.001
Beiderlinden et al. 2006 [72]	0.4 µg/kg/min	0.7 µg/kg/min	0.16
Brogan et al. 2009 [183]	57%	53%	0.16
Patroniti et al. 2011 [73]	61%	63%	n.s.
Schmidt et al. 2013 [137]	73%	66%	0.40
Azoulay et al. 2014 [113]	66.2%	76.6%	0.0004
Mendiratta et al. 2014 [58]	67%	72%	0.20
Wohlfarth et al. 2014 [76]	100%	100%	n.s.
Klinzing et al. 2015 [78]	54%	46%	0.81
Lee et al. 2016 [83]	73%	85%	0.321
Wohlfarth et al. 2017 [119]	71%	80%	0.63
Zayat et al. 2020 [184]	88.9%	88%	1.0

**Table 8:** Evidence for the use of vvECMO in obese patients and the respective outcome.

Study	BMI	ICU Mortality	Hospital Mortality	Odds Ratio
Al-Soufi et al. 2013 [189]	Quartile 1 Quartile 2 Quartile 3	n/a	33.2% 40.4% 33.3%	0.82 (0.60, 1.11) 0.93 (0.69, 1.25) 0.69 (0.50, 0.96)
Swol et al. 2014 [190]		50%	50%	1.05 (0.29, 3.77)
Lazzeri et al. 2016 [94]		31.5%	n/a	0.51 (0.26, 0.99)
Kon et al. 2015 [191]	<40 >40 >50	n/a	42% 33% 0%	n/a
Soubani et al. 2015 [192]	25–30 30–40 >40	n/a	n/a	0.89 (0.696, 1.13) 0.81 (0.62, 1.06) 1.1 (0.72, 1.695)
Lazzeri et al. 2017 [193]	25–30 30–40 >40	40.5% 28% 9.1%	n/a	0.41 (0.17, 1.01) 0.24 (0.08, 0.68) 0.06 (0.01, 0.47)
Swol et al. 2017 [194]	25–30 30–35 >35	38.7% 66.7% 52.4%	42% 78% 52.4%	1.01 (0.27, 3.78)/1.16 (0.31, 4.35) 3.20 (0.79, 13.02)/3.89 (0.94, 16.1) 1.60 (0.39, 6.63)/1.60 (0.39, 6.63)
Salna et al. 2018 [195]	All <30 30–40 >40	n/a	34.4% 33.6% 44.4% 17.9%	n/a
Keyser et al. 2020 [196]	<35	n/a	34%	n/a
Galvagno et al. 2020 [197]	25–30 30–35 35–40 >40	n/a	19.1% 32.7% 22.7% 19.5%	n/a

**Table 9:** Evidence for the use of vvECMO in trauma patients and the respective outcome.

Study	ICU Mortality	Hospital Mortality	Odds Ratio
Cordell-Smith et al. 2006 [220]	n/a	28.52%	n/a
Arlt et al. 2010 [161]	n/a	40%	n/a
Guirand et al. 2014 [210]	n/a	23.5%	n/a
Bosarge et al. 2016 [203]	n/a	13%	0.01 (0.06, 0.36)
Ull et al. 2017 [207] (review)	34.7%	30.6%	0.14 (0.06, 0.36)/0.22 (0.09, 0.52)
Grant et al. 2018 [204]	36%	45%	n/a
Strumwasser et al. 2018 [206]	n/a	60%	n/a
Ainsworth et al. 2018 [221]	29%	43%	n/a

## Data Availability

Reasonable requests with respect to data availability can be directed to the corresponding author.

## Supplementary Materials

The following are available online at https://www.mdpi.com/article/10.3390/membranes11080584/s1: Table S1. Evidence for the differences in SAPS II scores between the groups of survivors and non-survivors; Table S2. Evidence for the differences in SOFA scores between the groups of survivors and non-survivors; Table S3. Evidence for the differences in PRESERVE scores between the groups of survivors and non-survivors; Table S4. Evidence for the differences in RESP scores between the groups of survivors and non-survivors.
