# Supplementary material for: Contraindications to the Initiation of Veno-Venous ECMO for Severe Acute Respiratory Failure in Adults: A Systematic Review and Practical Approach Based on the Current Literature
Source: Membranes (Basel). 2021 Jul 30;11(8):584. doi: 10.3390/membranes11080584 (PMC8400963; doi:10.3390/membranes11080584)
Supplement: Supplementary file 1 [file membranes-11-00584-s001.zip › membranes-1248916-supplementary.pdf]

Contraindications to the Initiation of Veno-Venous ECMO for Severe Acute Respiratory Failure in Adults: A Systematic Review and Practical Approach Based on the Current Literature

Lars-Olav Harnisch * and Onnen Moerer

Department of Anesthesiology, University Medical Center, University of Goettingen, 37075 Göttingen, Germany; omoerer@med.uni-goettingen.de
* Correspondence: lars-olav.harnisch@med.uni-goettingen.de

Table S1. Evidence for the differences in SAPS II scores between the groups of survivors and non-survivors. Score values are absolute values, p-value as extracted from the respective trial. The cumulative mean value was not statistically significant between the two groups (Fishers' exact test).

Study	SAPS II Score Survivors	SAPS II Score Nonsurvivors	p-value
Beiderlinden et al. 2006	44	48	0.001
Schmidt et al. 2013	57	64	0.04
Wohlfahrt et al. 2014	56	45	0.90
Klinzing et al. 2015	43	51	0.09
Lee et al. 2016	50.35	68.7	0.0000
Choi et al. 2017	73	94	0.008
Wohlfahrt et al. 2017	56	55	0.61
Hilder et al. 2017	60	65	0.009
Stecher et al. 2018	62	69	0.27
Cho et al. 2019	54	67	0.043
Park et al. 2021	42	52.2	0.565
Supady et al. 2021	34	38.5	0.042
Fisser et al. 2021	61	64	<0.001
Giraud et al. 2021	56	60	NS
Mean	53.16	59.49	1.0

Table S2. Evidence for the differences in SOFA scores between the groups of survivors and non-survivors. Score values are absolute values, p-value as extracted from the respective trial. The cumulative mean value was not statistically significant between the two groups (Fishers' exact test).

Study	SOFA (mSOFA) Score Survivors	SOFA (mSOFA) Score Nonsurvivors	p-value
Beiderlinden et al. 2006	11 (7)	12 (8)	0.15
Arlt et al. 2010	12 (8)	12 (8)	n/a
Patroniti et al. 2011	7 (3)	10 (6)	<0.05
Schmidt et al. 2013	12 (8)	13 (9)	0.13
Enger et al. 2014	11 (7)	13 (9)	0.001
Wohlfahrt et al. 2014	12 (8)	12 (8)	0.79
Klinzing et al. 2015	11 (7)	12 (8)	0.09
Cheng et al. 2016/2	9 (5)	10 (6)	0.004
Choi et al. 2016	10 (6)	12 (8)	0.049
Lee et al. 2016	11 (7)	13 (9)	0.031
Serpa Neto et al. 2016	10.2 (6.2)	11.6 (7.6)	0.002
Wu et al. 2016	10 (6)	12 (8)	0.044
Hilder et al. 2017	13 (9)	15 (11)	<0.001
Hsin et al. 2016	9 (5)	10 (6)	0.005
Huang et al. 2016	10 (6)	12 (8)	0.238
Choi et al. 2017	14 (10)	17 (13)	0.022
Wu et al. 2017	9 (5)	10 (6)	0.002
Schmidt et al. 2018	13 (9)	13 (9)	0.83
Stecher et al. 2018	15 (11)	15 (11)	0.44
Cho et al. 2019	14 (10)	15 (11)	0.278
Park et al. 2021	9 (5)	12 (8)	<0.001
Supady et al. 2021	9 (5)	9 (5)	0.045
Fisser et al. 2021	12 (8)	13 (9)	0.011
Dreier et al. 2021	10 (6)	12 (8)	0.039
Mean	10.79 (6.79)	12.20 (8.20)	0.004

Table S3. Evidence for the differences in PRESERVE scores between the groups of survivors and non-survivors. Score values are absolute values, p-value as extracted from the respective trial. The cumulative mean value was not statistically significant between the two groups (Fishers' exact test).

Study	PRESERVE Score Survivors	PRESERVE Score Nonsurvivors	p-value
Hilder et al. 2017	4	5	0.106
Kang et al. 2017 (hospital)	5.6	6.9	0.021
Kang et al. 2017 (6 months)	5.4	7.0	0.007
Schmidt et al. 2018	6	8	0.001
Supady et al. 2021	4	3	0.389
Fisser et al. 2021	3	4	<0.001
Dreier et al. 2021	4	4	1.0
Mean	4.6	5.4	1.0

Table S4. Evidence for the differences in RESP scores between the groups of survivors and non-survivors. Score values are absolute values, p-value as extracted from the respective trial. The cumulative mean value was not statistically significant between the two groups (Fishers' exact test).

Study	RESP Score Survivors	RESP Score Nonsurvivors	p-value
Cheng et al. 2016	2	0	<0.001
Choi et al. 2016	1.31	−1.07	0.02
Hsin et al. 2016	2	−1	0.004
Wu et al. 2016	1	1	0.058
Hilder et al. 2017	1	−2	0.012
Kang et al. 2017 (hospital)	0.5	−2	0.004
Kang et al. 2017 (6 months)	0.5	−2	0.005
Wu et al. 2017	2	1	0.05
Schmidt et al. 2018	1	−1	0.002
Cho et al. 2019	2	1	0.703
Posluszny et al. 2020	−0.31	−0.83	<0.001
Supady et al. 2021	1	2	0.051
Fisser et al. 2021	0	−1	0.008
Mean	1.08	−0.45	0.432
